# Neonatal video review practices across the US: capturing utilization and barriers

**DOI:** 10.1038/s41372-026-02733-7

**Published:** 2026-06-01

**Authors:** M. E. Hill, A. M. Ades, E. E. Foglia, K. T. Wild, H. Berbari, B. A. Johnson, H. M. Herrick

**Affiliations:** 1 Division of Neonatology, Perinatal Institute, Department of Pediatrics, Cincinnati Children’s Hospital Medical Center, University of Cincinnati College of Medicine, Cincinnati, OH USA; 2 Division of Neonatology, Department of Pediatrics, Children’s Hospital of Philadelphia, Perlman School of Medicine, Philadelphia, PA USA; 3 House Staff, Department of Pediatrics, Cincinnati Children’s Hospital Medical Center, Cincinnati, OH USA

**Keywords:** Paediatrics, Health services

## Introduction

Clinical video review (VR) offers performance improvement opportunities through objective assessment of technical and nontechnical skills. VR has been used to assess Neonatal Resuscitation Program (NRP) compliance, drive quality improvement (QI), and evaluate delivery room educational initiatives [1, 2]. Despite the established benefits of VR for research, QI, and education, the national neonatal utilization of VR is unknown. We aimed to determine VR utilization and barriers for neonatal clinicians.

## Subjects and methods

### Study design

This is a descriptive observational study employing a survey created by resuscitation and VR experts. The survey was piloted and refined before implementation to ensure face validity. Survey questions included VR utilization, barriers to implementation, and technical and legal considerations (supplemental materials). Cincinnati Children’s Hospital Institutional Review Board deemed this study exempt (IRB ID 2024-0119).

### Study setting and population

The survey was sent to neonatal perinatal medicine fellowship program directors (PDs) within the United States identified through the American Academy of Pediatrics website (aap.org). Contact information was accessible for 95 of 108 fellowship PDs. Fellowship PDs were chosen because their contact information was readily available, and we were interested in understanding VR utilization at academic centers with trainees.

### Study protocol

These 95 PDs were invited to participate via email with a personalized link to a REDCap survey. The email contained an introduction to the study as well as consent to participate. Survey reminders were sent up to 6 times.

### Measurements and data analysis

Deidentified survey responses were analyzed. Descriptive statistics summarized data using mean and standard deviation or median and interquartile range as appropriate for continuous variables and percentages for categorical variables.

## Results

Between August 2024 and March 2025, 52/95 PDs (55%) completed the survey. Only 8 of the 52 responding programs (15.4%) use VR clinically. Table 1 highlights characteristics of the clinical VR programs among 8 fellowship programs across 9 hospitals (1 program video records at 2 hospitals). The most common location for VR is the delivery room (9/9; 100%); only 2 hospitals (2/9; 22.2%) utilize VR in the Neonatal Intensive Care Unit (NICU). The majority of delivery room VR is within dedicated infant resuscitation rooms (6/9; 66.7%). VR is more common in caesarian delivery rooms (4/9; 44.4%) as compared to labor and delivery rooms (2/9; 22.2%). The most common VR applications are QI (66.7%), clinical review debriefs/conferences (77.8%) and education (77.8%), with interprofessional teams the most common educational target audience (85.7%). Among the 9 hospitals with clinical VR, 8 (88.9%) have legal team involvement and all (100%) restrict access to VR recordings.

**Table 1:** Characteristics of clinical video review at 9 hospitals.

Characteristic	*n* (%)
Location of VR	
One Location	7 (77.8%)
Multiple Locations	2 (22.2%)
*Delivery Room:*^a^	**9 (100%)**
Level III Delivery Room	5 (55.6%)
Level IV Delivery Room	4 (44.4%)
*Within NICU:*	**2 (22.2%)**
Level III NICU	1 (11.1%)
Level IV NICU	1 (11.1%)
VR Equipment Type	
Permanent only	3 (33.3%)
Mobile only	4 (44.4%)
Both permanent and mobile	2 (22.2%)
VR Components^b^	
View of Resuscitation Bed	9 (100%)
View of Team	4 (44.4%)
Audio	9 (100%)
Vital Sign Monitoring	4 (44.4%)
Video Laryngoscope	1 (11.1%)
VR Applications^b^	
Clinical Review (Debriefs/Conferences)	7 (77.8%)
Quality Improvement	6 (66.7%)
Research	3 (33.3%)
Education	7 (77.8%)
*Educational Audience (n* = *7)*^b^	
Interprofessional Teams	6 (85.7%)
Fellows	3 (42.9%)
Residents	1 (14.3%)
Nurses	1 (14.3%)
Access to VR	
Restricted via Hospital Server	7 (77.8%)
Restricted via Private Computer	2 (22.2%)
Legal Team Involvement	
Yes	8 (88.9%)
Unsure	1 (11.1%)
Patient Consent	
Required	4 (44.4%)
Not required, patients cannot opt out	3 (33.3%)
Not required, patients can opt out	2 (22.2%)

*VR* video review, *NICU* neonatal intensive care unit.

^a^Including labor and delivery rooms, cesarian delivery rooms, and separate resuscitation rooms.

^b^Could select more than one response.

Among the 44 programs not using VR clinically, 2 are developing a VR program (4.5%), 4 previously used VR (9.1%), and 9 would like to implement VR but cannot due to barriers (20.5%). Among these 9 respondents interested in VR, identified barriers include: legal support (8; 88.9%), hospital administration (7; 77.8%), technology (6; 66.7%), cost (5; 55.6%), staff support (4; 44.4%), and leadership support (1; 11.1%).

Notably, clinical VR programs exist elsewhere in the hospital (ex. trauma bay) at 50% of programs (4/8) with VR and only 6.8% of programs (3/44) without VR. Furthermore, video is used in simulation by 25% of programs (2/8) with VR and 34% of programs (15/44) without VR.

## Discussion

We found clinical VR is rare in neonatology, with only 15% of respondent programs using clinical VR. This is comparable to 20% of surveyed adult and pediatric trauma centers, demonstrating VR underutilization extends beyond neonatology in medicine [3].

Most hospitals with clinical VR programs use it in the delivery room for clinical review and education, consistent with applications of VR outside of neonatology [4]. Interprofessional teams were the most common educational target, consistent with educational trends promoting interprofessional team collaboration and education both within and outside of VR education [5, 6].

Despite the proven benefits of VR, barriers hinder broader adoption. Programs reported legal team discomfort and lack of hospital leadership/staff buy-in as barriers to implementation. Partnership with legal teams and restricted access to videos is almost universal among hospitals with clinical VR programs. Additionally, clinical VR programs exist elsewhere in the hospital (ex. trauma bay) at 50% of programs (4/8) with VR and only 6.8% of programs (3/44) without VR. This suggests that a local precedent for VR and a preexisting partnership with legal and hospital administration may facilitate successful VR implementation.

## Conclusion

This study highlights the minimal national utilization of VR among academic neonatology divisions. Future efforts should focus on addressing barriers through open communication of shared VR experiences and procedures to increase use of this valuable tool. Programs with clinical VR should collaborate to spread the benefits of VR for education, research, and QI.

## Supplementary information

Video Review Survey

## Data Availability

De-identified data will be made available upon request after institutional data-sharing agreements are in place.
