# Supplementary material for: Neonatal video review practices across the US: capturing utilization and barriers
Source: J Perinatol. 2026 Jun 1;46(8):1550–2. doi: 10.1038/s41372-026-02733-7 (PMC13472870; doi:10.1038/s41372-026-02733-7)
Supplement: Supplementary file 1 [file 41372_2026_2733_MOESM1_ESM.pdf]

Video Recording Practices of Neonatology Divisions

Please complete the survey below.

Thank you!

Our Neonatology Division currently uses video recording of clinical care in at least one of our Neonatal Intensive Care Units (NICUs) and/or delivery rooms.	◯ Yes<br>◯ No
Our Division (select all that apply):	☐ Previously, but no longer uses video.<br>☐ Would like to use video, but cannot due to barriers.<br>☐ Is currently in the process of developing and/or implementing a video program.<br>☐ Uses video for simulation, but not for clinical care.<br>☐ Does not use video, but our institution uses video recording of clinical care in another area of the hospital.<br>☐ Does not use video, and it is not used elsewhere at our institution.
Why not?	__________________________________
Our barriers are (select all that apply):	☐ Legal<br>☐ Hospital Administration<br>☐ Technology<br>☐ Cost<br>☐ Staff Support<br>☐ Leadership Support<br>☐ Other
Other barriers (free text):	__________________________________
Video recording is used elsewhere in the hospital in the (select all that apply):	☐ Trauma Bay/Emergency Department<br>☐ Pediatric Intensive Care Unit (PICU)<br>☐ Cardiac Intensive Care Unit (CICU)<br>☐ Operating Room<br>☐ Other<br>☐ Not sure
Other areas (free text):	____________________________
Our Division uses video recording of clinical care in the following areas (select all that apply):	☐ Delivery Room setting at a Level III NICU<br>☐ In the unit at a Level III NICU<br>☐ Delivery Room setting at a Level IV NICU<br>☐ In the unit at a Level IV NICU<br>☐ Delivery Room setting at a Level II NICU<br>☐ In the unit at a Level II NICU
For delivery room resuscitations, we video record in:	☐ Labor and delivery rooms<br>☐ Operating rooms<br>☐ Dedicated infant resuscitation rooms

Which L&D rooms?	◯ Some rooms<br>◯ All rooms
In the delivery room, we video record:	◯ Every resuscitation<br>◯ Resuscitations that meet certain criteria<br>◯ Resuscitations at our discretion
In the unit, we video record:	☐ Every code<br>☐ Every procedure<br>☐ Codes and/or procedures that meet certain criteria<br>☐ Codes and/or procedures at our discretion
Describe the criteria (both for in-unit and delivery room recordings where applicable):	__________________________________
Describe what "at your discretion" means to you (both for in-unit and delivery room recordings where applicable). What does your division choose to record?	__________________________________
Our institution video records clinical care elsewhere in the hospital in the (select all that apply):	☐ Trauma Bay/Emergency Department<br>☐ Pediatric Intensive Care Unit (PICU)<br>☐ Cardiac Intensive Care Unit (CICU)<br>☐ Surgical Operating Room (NOT referring to C-sections)<br>☐ Other<br>☐ Not sure<br>☐ It is NOT used elsewhere in the hospital
Other areas (free text):	____________________________
Our Division/NICU reviews videos for the following purposes (select all that apply):	☐ Clinical review such as debriefs or conferences (example: entire code/delivery team watches and debriefs resuscitation to identify ways to improve both clinical/team performance)<br>☐ Education (examples: use of videos during didactics, use of videos in orientation as "gold standard" examples)<br>☐ Documentation as part of the permanent patient record<br>☐ Standardized data collection<br>☐ Research projects<br>☐ Quality improvement projects<br>☐ Simulations<br>☐ Review with parents<br>☐ Other
Describe in more detail how you use video for clinical review and/or education (hot debriefs, cold debriefs, conferences, didactics, etc- the more detail the better):	__________________________________

Our educational sessions target (select all that apply):	☐ The entire interprofessional team as a group (nurses, respiratory therapists, physicians, etc.)<br>☐ The NICU Fellows separately<br>☐ The Pediatric Residents separately<br>☐ The Advanced Practice Providers separately (Nurse Practitioners, Physician Assistants, etc.)<br>☐ Physicians separately<br>☐ Nurses separately
Please explain in as much detail as possible how video is used for each group of learners:	__________________________________
Who reviews the videos to abstract/collect the data?	__________________________
Other purpose of video review (free text):	__________________________________
What video recording system do you use?	__________________________________
Our equipment is (select all that apply):	☐ Permanently installed<br>☐ Mobile equipment
How many cameras are set-up for recordings with your permanently installed equipment?	__________________________
Please describe the views captured by these cameras (example: overhead view of the baby AND global view of the entire team):	__________________________________
How many cameras are set-up for recordings with your mobile equipment?	__________________________
Please describe the views captured by these cameras (example: zoomed out view of the entire team):	__________________________________
Video recordings have accompanying (select all that apply):	☐ Audio<br>☐ Vital sign monitor data<br>☐ Additional physiologic data (example: respiratory function monitor)<br>☐ Video laryngoscope input<br>☐ Other
Other (free text):	__________________________
Are you required (legally or otherwise) to review and/or abstract data from video recordings?	◯ Yes<br>◯ No
We are required to review and/or abstract data from:	◯ Every video recording that has been generated<br>◯ Only video recordings that meet certain criteria

Describe the criteria: __________________________________

Is patient (parent/legal guardian) consent needed to generate a video recording?

- ◯ Yes and must be signed BEFORE the recording is generated
- ◯ Yes but can be signed AFTER the recording is generated
- ◯ No but patients can opt out
- ◯ No and patients cannot opt out
- ◯ Not sure

How is patient consent obtained?

- ◯ Part of the general admission and consent to treat form
- ◯ Separate written consent for video recording
- ◯ Verbal consent/assent
- ◯ Other

Describe how patient consent is obtained: __________________________________

Is provider (nursing, physician, etc.) consent needed for video recordings?

- ◯ Yes and must be signed BEFORE the recording is generated
- ◯ Yes but can be signed AFTER the recording is generated
- ◯ No but providers can opt out
- ◯ No and providers cannot opt out
- ◯ Not sure

How is provider consent obtained?

- ◯ Written consent for video recording
- ◯ Verbal consent/assent
- ◯ Other

Describe how provider consent is obtained: __________________________________

A separate consent is needed (from patients and/or providers) to USE a video recording for (select all that apply):

- ☐ Debriefs internally at your own institution
- ☐ Formal presentations internally at your own institution
- ☐ Formal presentations externally outside your institution
- ☐ Creation of permanent educational resources to be used internally
- ☐ Creation of permanent educational resources to be used externally
- ☐ QI Projects
- ☐ Research studies
- ☐ Not sure

Notice of video recordings is posted in the area where recordings occur:

- ◯ Yes
- ◯ No
- ◯ Not sure

Is access to video recordings restricted?

- ◯ Yes
- ◯ No

Who has access?	__________________________________
How/where do you have access?	◯ Hospital server (in-hospital only)<br>◯ Hospital server (with remote access possible, including on personal computer with VPN)<br>◯ Private work computer (but not connected to hospital server)<br>◯ Personal computer (not requiring hospital server/VPN access)
Is storage of video recordings limited?	◯ Yes<br>◯ No
Storage is limited by (select all that apply):	☐ A pre-specified length of time<br>☐ Server capacity<br>☐ Leadership/provider discretion
How long are videos stored?	____________________________
The legal team at our institution is involved in video recording program policy and oversight:	◯ Yes<br>◯ No<br>◯ Not sure
Video recordings have been requested for and/or used in legal proceedings:	◯ Yes<br>◯ No<br>◯ Not sure
Parents can request a copy of the video recording:	◯ Yes<br>◯ No<br>◯ Not sure
Name of Person Completing the Survey	____________________________
Academic Title of Person Completing the Survey	____________________________
If applicable, please describe your personal role/involvement in your Division's video review program:	____________________________
Email of Person Completing the Survey	____________________________
Name of Neonatal-Perinatal Medicine Fellowship Program	____________________________
Total number of fellows in your program	____________________________
